# Parental psychosocial factors associated with parental reporting of their child’s administrative ADHD diagnosis - results from the consortium project INTEGRATE-ADHD

**DOI:** 10.1186/s12888-025-07575-9

**Published:** 2025-11-05

**Authors:** Ann-Kristin Beyer, Lilian Beck, Heike Hölling, Thomas Jans, Sophia Weyrich, Marcel Romanos, Anne Kaman, Ulrike Ravens-Sieberer, Julian Witte, Peter Heuschmann, Cordula Riederer, Robert Schlack

**Affiliations:** 1 Department of Epidemiology and Health Monitoring, Robert Koch Institute, Gerichtstraße 27, 13347 Berlin, Germany; 2 Department of Child and Adolescent Psychiatry, Psychosomatics and Psychotherapy, Centre of Mental Health, University Hospital Würzburg, Würzburg, Germany; 3 Department of Child and Adolescent Psychiatry and Psychotherapy, Research Section “Child Public Health”, University Medical Centre Hamburg-Eppendorf, Hamburg, Germany; 4 Vandage GmbH, Bielefeld, Germany; 5 Institute of Clinical Epidemiology and Biometry, Julius Maximilian University of Würzburg, Würzburg, Germany; 6 Clinical Trial Centre, University Hospital Würzburg, Würzburg, Germany; 7 Institute for Medical Data Sciences, University Hospital Würzburg, Würzburg, Germany; 8 DAK-Gesundheit, Hamburg, Germany

**Keywords:** Routine data, Survey data, Child ADHD diagnosis, Parental reporting, Psychosocial, Parental strain, Parental ADHD, Parental psychological problems, Family cohesion, Health literacy

## Abstract

**Background:**

As one of the most commonly diagnosed psychiatric disorders in children and adolescents, reliable prevalence data on attention-deficit/hyperactivity disorder (ADHD) is highly relevant to health policy and health care planning. However, routine data and parental diagnosis reports from surveys − as important data sources on child ADHD − often differ. This study investigates whether parental psychosocial factors are associated with parental diagnosis reporting in German parents whose child is registered with an administrative ADHD diagnosis (ICD-10 F90.0-9) with their statutory health insurance. We expected more parental burden to be associated with a lower likelihood of a parental diagnosis report.

**Methods:**

Parents of 5,461 children and adolescents who presented with an administrative ADHD diagnosis in 2020 answered online questions about their child’s ADHD diagnosis and various psychosocial characteristics, including parental strain, parental psychological problems, parental ADHD diagnosis, family cohesion and parental health literacy. Chi-square tests and unadjusted linear regressions were used to analyze group differences in parental psychosocial characteristics between parents who reported the ADHD diagnosis and those who did not. Binary logistic regressions were conducted to predict the parental report of their child’s ADHD diagnosis in the survey.

**Results:**

Group comparisons revealed that parents who reported their child’s ADHD diagnosis displayed significantly more parental strain, more psychological problems, higher rates of maternal and paternal ADHD, lower levels of family cohesion and lower health literacy than parents who did not report their child’s ADHD diagnosis. The results were partly confirmed in multivariate analysis, where maternal (OR = 3.18) and paternal ADHD (OR = 2.94) turned out to be the strongest predictor of a parental diagnosis report.

**Conclusions:**

Contrary to our expectations, parental psychosocial burden, in particular parental ADHD diagnosis, increased the likelihood of a parental report of their child’s ADHD diagnosis, which may point to a greater sensitivity and awareness of affected parents towards their child’s ADHD. The findings suggest that differences in the diagnosis prevalence of child ADHD between routine and survey data may vary as a function of parental psychosocial factors.

## Introduction

Attention-deficit/hyperactivity disorder (ADHD) is a highly prevalent psychiatric disorder among children with core symptoms of inattention, hyperactivity and impulsivity [1]. The worldwide-pooled prevalence of ADHD among children and adolescents is estimated at around 5% [2, 3]. ADHD often persists into adulthood, and negatively influences the educational, behavioral, emotional and social functioning as well as the health-related quality of life [4–8] of those affected and is associated with an increased risk of accidents, criminality or suicidal behavior [9–11]. In addition, psychiatric comorbidity is high [12–15]. A recent claims data analysis showed 2.86-fold higher annual health care costs for children and adolescents with an incident ADHD diagnosis compared to a reference group consisting of children and adolescents without ADHD [16]. In sum, the disorder may tremendously burden the affected individuals, their families as well as the healthcare system and society.

Reliable figures on the prevalence of ADHD are therefore indispensable for health policy and health care planning. However, population-based prevalence rates vary considerably depending, amongst others, on the data used. For example, while prevalence figures of child ADHD in German statutory health insurance data increased considerably over the past two decades [17–19], prevalence rates in epidemiological studies, based on parents’ reports of an ADHD diagnosis made by a doctor or psychologist, remained stable over the same period, or even declined [20, 21]. Diagnosis prevalences and temporal diagnosis trends varying in this way must necessarily lead to varying conclusions.

To better understand potential causes of such discrepancies, we carried out the project INTEGRATE-ADHD [22, 23]. In the framework of this project, parents of youths who were insured with the third largest German statutory health insurance and who had an administratively documented ADHD diagnosis were surveyed online on health issues, amongst others on their child’s ADHD diagnosis. A core finding of this project was, that only 71.6% of the interviewed parents reported their child’s administrative ADHD diagnosis in the survey while almost one third of the parents did not [24]. In this publication, we also examined the distribution of the parental reporting behavior as a function of various sociodemographic variables. Amongst others, we found that the parents of girls, younger children and youths with migration background were less likely to report the ADHD diagnosis of their child. We also conducted a series of utilization-based analyses to explore the possible reasons for the parental reporting behavior [25]. For example, we found that although most of the child ADHD diagnoses were made in pediatric care, only diagnoses made in psychiatric-psychotherapeutic care were associated with the parental diagnosis report in multivariate analysis. The results suggested problems in doctor-patient communication, in particular in pediatric care [25]. Both investigations pointed to problems in doctor-patient communication as one of the prerequisites for a parental reporting their child’s diagnosis in a survey is that parents had been adequately informed about the diagnosis. The diagnosis must thus not only have been communicated at all, but also in a way that is understandable and comprehensible to the parents.

In addition, parents must be willing to report their child’s diagnosis when being asked. One reason why parents may not report their child’s ADHD diagnosis may be that they were psychosocially too burdened. Everyday life with a child with ADHD can be challenging. Parents of children with ADHD experience increased strain and stress levels [26, 27]. Moreover, families with a child with ADHD are more likely to experience a greater amount of family conflicts, lower family cohesion, and in general a less organized family life due to the child’s ADHD [28, 29]. Because of their increased strain and daily hazzles, parents might be less likely to report their child’s ADHD diagnosis in a survey. Additionally, it can be assumed that parents displaying a lower health literacy might be less able to follow communication of the diagnosis by professionals, which may in turn be associated with a lower likelihood of a parental diagnosis reporting. Further, a meta-analysis revealed that parents of children with ADHD displayed higher rates of psychological problems than parents of children without ADHD [30] or, given the high heritability of ADHD, were affected themselves by the disorder [31]. Everyday challenges to provide and maintain a structured daily life may then rise significantly [32, 33]. On the other hand, parents with ADHD may also perceive greater levels of tolerance or empathy towards their child’s behavior given their own experience with the disorder and might think that the child is just like the father or mother. Parents might thus not classify their child’s behavior as pathologic and/or might not accept a doctor’s or psychologist’s ADHD diagnosis and, as a consequence, might not report it when being asked about it.

The primary goal of this study is to explore associations of parental psychosocial factors with the parental reporting of their child’s ADHD diagnosis. We expect that more parental psychosocial burden in terms of more strains, psychological problems, the presence of parental ADHD as well as lower family cohesion and health literacy is associated with a lower likelihood of a parental report of their child’s administratively documented ADHD diagnosis. To the best of our knowledge, this is the first attempt to address the question whether and to what extent parental psychosocial burdens are associated with parents’ reporting behavior in an epidemiological survey. The results may provide valuable insights into the validity of parental diagnosis reports and help to clarify discrepancies between routine data and epidemiological survey data.

## Methods

### Study design and response

Within the framework of the consortium project INTEGRATE-ADHD a cross-sectional online survey was conducted from October 2021 to August 2022. Out of a source population of 848,110 children and adolescents insured with Germany’s third largest nationwide operating statutory health insurance, DAK-Gesundheit, a total of 24,880 parents of children fulfilling the following inclusion criteria: child was (1) insured with DAK-Gesundheit in 2020, (2) 0 to 17 years old in 2020, and (3) registered with an administratively documented ADHD diagnosis (ICD-10 F90.0-9, labelled as ‘confirmed’) in at least one quarter of 2020 (so-called M1Q criterion), were invited to take part in the survey (gross sample). A total of 5,461 parents answered the online questionnaire (net sample), which corresponds to a response rate of 21.5% according to the American Association for Public Opinion Research (AAPOR) Standard Definitions, Version 9 (RR3) [34]. For more details on study design, sampling and response see Beyer et al. [23].

### Instruments

In the online survey, modified questionnaires from the nationwide epidemiological German Health Interview and Examination Survey for Children and Adolescents (KiGGS study; [35–37]) and its in-depth module on mental health (BELLA study; [38, 39]) were deployed.

The *child’s ADHD diagnosis* was assessed asking the parents whether their child had ever been diagnosed with ADHD by a physician or a psychologist [20, 40].

*Parental strain* was assessed using the parental strain scale by Sperlich et al. [41]. This scale consists of 13 different types of parental strain, e.g. financial worries, parenting problems or conflicts with the partner, as well as daily stress in general. The severity of parental strain could be rated for each of the types on a five-point Likert scale ranging from 1 (not at all) to 5 (very strong). For the present analyses, the response options ‘strong’ and ‘very strong’ were pooled into ‘stressed’ according to Bolster et al. [42], the rest was pooled into ‘not stressed’. Finally, the strains were summed up across all 13 items and categorized into ‘none’, ‘1 parental strain’, ‘2 parental strains’, ‘3 parental strains’ and ‘4 and more parental strains’ [42].

*Paternal and maternal ADHD diagnosis* were assessed by asking the respondents whether the mother and the father had ever been diagnosed with ADHD by a physician or a psychologist.

*Parental psychological problems* were measured with the nine-item short version of the Symptom Checklist 90-R (SCL-K9; [43]) that had to be answered on a five-point Likert scale ranging from 0 (not at all) to 4 (extremely). The items were summed up to a total score ranging from 0 to 36, with higher scores indicating stronger psychological problems.

*Family cohesion* was assessed using a short form of the German Family Climate Scale [44]. The short form consists of nine items that had to be answered on a four-point Likert scale ranging from 1 (not true) to 4 (exactly true) [45]. The sum score was transformed into values between 0 and 100, with higher values indicating higher levels of family cohesion.

*Health literacy* was measured using the 16-item short version of the European Health Literacy Survey [46]. The respondents were asked about difficulties in the areas of accessing, understanding and using health information. The response options ranged from 1 (very simple) to 4 (very difficult). For analysis, a total score from 0 to 16 was calculated with higher values indicating higher levels of health literacy.

The following sociodemographic variables were used as *covariates* in multivariate analysis. More detailed results on the sociodemographic variation of the parental reporting behavior can be taken from Schlack et al. [24]. Age (in years) was included as age at the time of the survey, gender was included as binary variable (female/male). Parental education was categorized using the internationally comparable educational classification Comparative Analysis of Social Mobility in Industrial Nations (CASMIN; [47]). The classification includes information on both school education and vocational training with level 1 (low), 2 (medium) or 3 (high). A child was classified having a migration background if both parents or the child itself immigrated to Germany or if both parents did not hold German citizenship [48]. Family status was categorized as ‘nuclear family’ (child living with both biological parents), ‘single-parent family’ (child living with only one biological parent but without a partner) or ‘stepfamily’ (child living with one biological parent and a partner). All other forms of family living arrangements were pooled to ‘other’.

### Statistical analyses

Group comparisons between categorical variables were calculated using the Rao-Scott chi-square test. In the case of continuous variables, unadjusted linear regression modeling was used. To predict the likelihood of a parental report of the child’s ADHD diagnosis (yes/no), a binary logistic regression analysis was deployed including parental strain, parental ADHD, parental psychological problems, family cohesion and health literacy as independent variables as well as age, gender, parental education, migration background and family status as covariates. The statistical significance for all analyses was set to *p* < .05. Effect sizes are given as odds ratios (OR).

In order to adjust for deviations of the net sample from the gross sample, population weights were calculated that standardize the net sample to the gross sample [23]. The population weights are determined by the inverse probability of a person participating in the survey. People with a low probability of participation represent more people from the population than people with a high probability of participation. The subsequent analyses were performed on the weighted data. All analyses were performed with Stata version 17.0 using the svy-procedure for complex random samples.

## Results

### Descriptive statistics

Table 1 depicts the sample characteristics. Mean age of children and adolescents at the time of the survey was 12.6 years (*SE* = 0.05, range 2–19 years), 74.1% were male. The majority of the parents displayed a medium level of education (63.2%), most of the children had no migration background (93.5%) and about half of the children lived in nuclear families (55.3%).

**Table 1:** Sociodemographic characteristics of the sample

	*n*	%	(95% CI)
**Number of participants**	5,461		
**Age group** ^a^			
0–2 years	3	0.1	(0.0-0.2)
3–6 years	167	3.5	(3.0–4.0)
7–10 years	1,351	24.3	(23.1–25.4)
11–13 years	1,827	31.2	(30.0-32.5)
14–17 years	1,770	33.4	(32.2–34.8)
18–19 years	343	7.5	(6.8–8.3)
**Gender** ^a^			
Female	1,386	25.9	(24.7–27.1)
Male	4,075	74.1	(72.9–75.3)
**Parental education** ^a^			
Low	560	10.4	(9.6–11.3)
Medium	3,271	63.2	(61.9–64.5)
High	1,355	26.4	(25.1–27.6)
**Migration background** ^a^			
Migration background	332	6.5	(5.9–7.3)
No migration background	4,948	93.5	(92.7–94.1)
**Family status**			
Nuclear family	3,013	55.3	(54.0-56.7)
Single-parent family	1,048	19.5	(18.5–20.6)
Stepfamily	814	14.6	(13.7–15.6)
Other	585	10.6	(9.8–11.4)

Note. ^a^ already published in Schlack et al. [24]; *n* unweighted, % weighted; Numbers not summing up to total due to missing data; CI = Confidence interval

Table 2 shows the frequencies and mean values for parental psychosocial characteristics overall and by parental report of their child’s ADHD diagnosis. As already reported above, only 71.6% of parents had reported their child’s administrative diagnosis of ADHD in the survey [24]. On average, parents reported 2.64 daily strains. Parents who reported their child’s ADHD diagnosis indicated significantly more parental strains (*M* = 2.87) than parents who did not (*M* = 2.03; see Table 2). A proportion of 4.7% of the mothers and 5.0% of the fathers reported that they themselves had ever been diagnosed with ADHD. Parents who reported their child’s ADHD diagnosis had themselves ever been diagnosed with ADHD significantly more frequently ADHD than parents who did not report their child’s ADHD diagnosis (mothers: 6.1% vs. 1.5%, fathers: 6.2% vs. 2.3%). The average score of parental psychological problems was *M* = 8.51. Parents who reported their child’s ADHD diagnosis displayed significantly higher levels of psychological problems (*M* = 9.08) than those who did not (*M* = 7.08). The mean of the family cohesion scale was 61.17 and of the health literacy scale 13.61. Parents who reported their child’s ADHD diagnosis indicated significantly lower family cohesion and health literacy (*M* = 59.85 and *M* = 13.48, respectively) compared to those parents who did not (*M* = 64.51 and *M* = 13.93, respectively).

**Table 2:** Frequencies and mean values overall and by parental report of their child’s ADHD diagnosis for parental psychosocial characteristics

	Total *(n* = 5,211)		Parental diagnosis report *(n* = 3,947)		No parental diagnosis report (*n* = 1,264)		
	*n (%)*	*M (SE)*	*n (%)*	*M (SE)*	*n (%)*	*M (SE)*	*p*
**Number of parental strains** (*n* = 5,062)		2.64 (0.04)		2.87 (0.05)		2.03 (0.07)	< 0.001
**Maternal ADHD diagnosis** (*n* = 4,645)							<0.001
Yes	237 (4.7%)		218 (6.1%)		19 (1.5%)		
No	4,408 (95.3%)		3,270 (93.9%)		1,138 (98.5%)		
**Paternal ADHD diagnosis** (*n* = 3,971)							< 0.001
Yes	212 (5.0%)		186 (6.2%)		26 (2.3%)		
No	3,759 (95.0%)		2,724 (93.8%)		1,035 (97.7%)		
**Parental psychological problems** (*n* = 5,080)		8.51 (0.10)		9.08 (0.12)		7.08 (0.18)	< 0.001
**Family cohesion** (*n* = 5,170)		61.17 (0.24)		59.85 (0.27)		64.51 (0.48)	< 0.001
**Health literacy** (*n* = 5,037)		13.61 (0.04)		13.48 (0.05)		13.93 (0.08)	< 0.001

Note. *n* unweighted, % and *M (SE)* weighted; Numbers not summing up to total due to missing data

### Prediction of the parental report of the child’s ADHD diagnosis

Multivariate logistic regression analysis yielded significant associations of parental strain, parental ADHD and psychological problems as well as of lower family cohesion with a parental diagnosis report (see Table 3). The strongest associations were found for lifetime ADHD diagnosis of the mother (OR = 3.18) and the father (OR = 2.94). The presence of two parental strains was associated with a 1.39 times higher likelihood of a parent report, the presence of three strains increased the likelihood by a factor of 1.81, and the presence of four and more strains by a factor of 1.78. Parental psychological problems as measured with the SCL-K9 were significantly but only weakly associated (OR = 1.02). In contrast, a good perceived family cohesion significantly decreased the likelihood of a parental reporting of the child’s ADHD diagnosis (OR = 0.99). Health literacy was not significantly associated with parental diagnosis reporting.

**Table 3:** Weighted binary logistic regression analysis predicting the parental report of their child’s ADHD diagnosis (*n* = 3,747)

		AOR	95% CI	*p*
Number of parental strains	0	Ref		
	1	1.14	0.90–1.45	0.287
	2	**1.39**	**1.08–1.80**	**0.012**
	3	**1.81**	**1.36–2.41**	**< 0.001**
	4 and more	**1.78**	**1.40–2.25**	**< 0.001**
Maternal ADHD diagnosis	No	Ref		
	Yes	**3.18**	**1.84–5.48**	**< 0.001**
Paternal ADHD diagnosis	No	Ref		
	Yes	**2.94**	**1.88–4.59**	**< 0.001**
Parental psychological problems		**1.02**	**1.01–1.04**	**0.004**
Family cohesion		**0.99**	**0.99-1.00**	**0.002**
Health literacy		0.99	0.96–1.02	0.470

Note. AOR = adjusted odds ratio, adjusted for gender, age (years at the time of the survey), parental education, migration background and family status; CI = Confidence interval; bold print: significant at the *p* < .05 level

## Discussion

The aim of this study was to examine associations of various parental psychosocial characteristics with the parental report of their child’s administrative ADHD diagnosis. We assumed thereby that psychosocially burdened parents were less likely to report their child’s administrative ADHD diagnosis in the survey in comparison to non-burdened parents.

Contrary to this expectation, we found that parents who displayed significantly more strains, higher levels of psychological problems, higher rates of maternal and paternal ADHD, and lower levels of family cohesion were more likely to report their child’s ADHD diagnosis. A potential explanation may be that parents − unlike assumed − may have a greater awareness of their child’s ADHD when they more burdened by their child’s disorder and the accompanied challenges in everyday life, possibly because they pay more attention to their child’s behavior, interpret his or her behavior more likely as symptomatic, and therefore recall symptoms in the child better [49]. This may, in turn, increase their willingness to report their child’s ADHD diagnosis.

Child and parental ADHD, parental psychological problems and parental stress are often interdependent [27, 30, 50]. Parental psychological problems in general are associated with higher strain, more family conflict and lower family cohesion [28], and parents with ADHD display more difficulties in interacting with their child and experience greater parenting stress [51, 52]. The results show that in multivariate analysis, among all investigated parental psychosocial characteristics, parental ADHD stood out as the strongest predictor. Both maternal and paternal ADHD positively predicted a parental diagnosis report in a similar height. Unlike our initial assumption, parents who are themselves diagnosed with ADHD may be more sensitive and more aware towards the ADHD of their children, potentially because they experience similar challenges in their own and in their child’s lives. Parents with ADHD may also be more likely to seek help, may have had more frequently contact with the healthcare system, or already have experienced ADHD treatment themselves. Therefore, they might be more familiar with ADHD, which in turn may increase their awareness towards their child’s ADHD, their likeliness to accept it and their willingness to report its diagnosis in a survey. As indicated initially, there are some more possible explanations for the parental non-reporting of the child’s administrative ADHD diagnosis. Based on data from the project INTEGRATE-ADHD, we identified both in an analysis based on sociodemographic variation [24] and in an utilization-based analysis potential communication biases between health care providers and parents in the pediatric or GP setting [25]. The present study extends these findings by emphasizing the significance of parental psychosocial burden, in particular parental ADHD, for the willingness of parents to report their child’s ADHD diagnosis in a survey.

### Strengths and limitations

INTEGRATE-ADHD is the first study in Germany to allow simultaneous analyses of ADHD diagnostic data from administrative and survey origin linked at person level, which is a considerable strength. In addition, we were able to use a large and roughly representative sample for our analysis, which allows a good generalization of our findings [23]. Albeit weighting our analyses, however, we were not able to adjust for the specific “not missing at random” non-response of parents of children with ADHD who may not have participated because they were sure their child does have ADHD or who may not have participated because they were sure their child does not have ADHD. A further limitation is that the analyses are cross-sectional which does not allow the examination of causality. Another limitation may consist in the validity of the child’s administrative ADHD diagnosis and the quality of the diagnosis documentation by health care providers. To ensure that the administrative ADHD diagnoses were not unconfirmed or tentative, only those cases with confirmed ADHD diagnoses were included in the study. Albeit significantly negatively associated in bivariate analysis, health literacy was no longer significantly associated with a parental diagnosis report in multivariate analysis. In our study, we used a short questionnaire measuring general health literacy. A health literacy questionnaire specifically for mental health purposes or – in particular –specific for ADHD might even provide more nuanced results and reveal more differentiated associations with the parental report behavior. This could be the subject of further studies.

## Conclusions

In sum, we were able to show that the parental diagnosis reporting varies as a function of parental psychosocial factors. The findings thus highlight the contribution of parental psychosocial factors − in particular maternal and paternal ADHD − for a better understanding of parental reporting behavior in surveys and shed light on putative causes for diverging prevalences of ADHD diagnostic data from administrative and survey data. With regard to clinical practice, our results suggest that special communication efforts, e.g. in the form of a pronounced psychoeducation, are particularly necessary when parents appear less burdened and have not yet had any ADHD-specific contact with the healthcare system themselves.

## Data Availability

The datasets generated and analyzed during the current study are not publicly available due to access restrictions of the administrative data, which may not be made publicly accessible.

## Abbreviations

AAPOR: American Association for Public Opinion Research
ADHD: Attention-deficit/hyperactivity disorder
BELLA: Mental health module of KiGGS
CASMIN: Comparative Analysis of Social Mobility in Industrial Nations
ICD: International Statistical Classification of Diseases and Related Health Problems
KiGGS: German Health Interview and Examination Survey for Children and Adolescents
SCL-K9: Short version of the Symptom Checklist
